# Medical Students:—A New Generation

**Published:** 1861-10

**Authors:** 


### Art. VIII.—MEDICAL STUDENTS:—A NEW GENERATION.

A republication of "The London Medical Student," a brochure from the pen of the late Mr. Albert Smith, and originally contributed as a serial story to *Punch* in its early days, induces us to cast a glance at the character and habits of the young men by whom the ranks of the profession are recruited. In times past they have been made the sport of fiction, and it is due to them to show how little that fiction can be justified.

We are convinced that Mr. Smith, although himself a member of the medical profession, did not draw from observation the characters that he describes. His medical students are clearly hypothetical animals, imagined under the impression that the rules and regulations of the licensing bodies of his day were strictly carried out, and that they produced infallibly their natural results. So far was this from being the case, that, in spite of the influence of the Apothecaries' Company, the medical students of London have for many years been constantly increasing in steadiness, diligence, and self-respect.

The error into which Mr. Smith's book is calculated to lead those who may peruse it, is very pardonable in an unprofessional writer. We can smile at the "Sawbones in training" of Mr. Dickens, and sympathize with the enthusiasm with which Mr. Pickwick, prior to experience, felt and expressed concerning them. But they are not brought before us as average specimens of the class to which they belonged, neither are they held up as examples for imitation. Their faults are redeemed by an admixture of humour and kindliness, and their characters early display a sufficiency of sense to form a probable basis for their eventual reformation. From Mr. Muff and his friends, however, the heroes of Mr. Smith's book, we turn with unmixed disgust; their acquired profligacy in no way redeeming the dulness of their natural imbecility, and the whole production exciting our surprise that any writer of repute could attempt to foist such creations upon the public. *Ex nihilo nihil*, and the general practitioners of England, with all their faults and shortcomings, possess qualities of heart and mind for which Mr. Smith gives them no credit, and an amount of skill and knowledge such as no amount of "grinding" could supply.

Until quite recently, however, medical education was conducted under regulations which were exactly calculated to make students all that Mr. Smith describes, and which, notwithstanding that they were largely evaded by the common sense of the public, have set their seal of evil on the profession. The Act of 1815 gave a practical monopoly of middle-class medical education to the Apothecaries, a trading company, whose corporate mind returned continually to the contemplation of rhubarb, and whose members regarded the preparation and sale of physic as the highest possible achievement of their art. In order that pills might be perfectly round, they condemned their future licentiates to suffer a five years' apprenticeship; and bowing to the well-known dictum that the compounding of medicines was an employment unworthy of gentlemen of liberal education, they took care to frame such regulations that no licentiate of theirs should ever presume to be liberally educated. For the attainment of this end, they gave the licence at the age of twenty-one years; and prescribed a hospital course and an apprenticeship, extending together over eight years, so as to necessitate removal from school at the age of thirteen. It is evident that a boy of thirteen cannot usually have made sufficient progress in the great essentials of education to be at all under their influence in point of intellectual character. Even in the most favourable cases, he will have done little more than to overcome the preliminary difficulties of acquisition—the mere drudgery of learning; and if he is there to stop, he might have been employed with equal advantage to his future career upon drudgery of any other kind. It is also evident that no prudent practitioner would allow an apprentice of thirteen to compound medicines; and that no prudent patient would swallow anything suspected to be such youthful handiwork. Practically, therefore, the first years of apprenticeship were spent in merely menial drudgery, in the tasks and duties of an errand-boy. Washing bottles, carrying out medicines, opening doors, running errands, and inventing evasive answers in his master's absence; such were the ordinary occupations of the young apprentice. As time passed on a larger sphere of action opened to him. "To see practice" became an object of ambition—attainable only by dint of practising upon the poor. And masters who reviled druggists for counter practice, who revelled in visions of some distant future in which all quacks should be whipped after a summary conviction, who believed that the sale of a pennyworth of jalap ought to be a penal offence, and that protection for the British doctor would be the *summum bonum* of judicious legislation, these very masters permitted their apprentices to visit and prescribe while in utter ignorance of the veriest rudiments of pathology and therapeutics.

This "seeing of practice," in large towns especially, involved another evil—that of attendance upon labours. The young apprentices were to be encouraged to soothe the throes of the parturient prostitute, and to defile their adolescence with all the filthy associations by which low midwifery is surrounded, with the companionships, the conversation, and the gin.

As a finale to these proceedings, the emancipated apprentice was to settle in some large town, there to join a Medical School in the capacity of a full-blown student, and to attend lectures upon many profound and difficult subjects. It is hardly surprising, we repeat, that writers of fiction should have imagined his moral and intellectual status to be such as the ordeal we have described seems calculated to produce, or that they should have delineated their typical student as an imbecile and a hog. In doing so, however, they neglected to take into account certain sources of compensation, and hence have produced a caricature instead of a resemblance.

For experience has shown that the common sense of mankind early rose up against the yoke of the Worshipful Company, and contrived to keep within the letter, while violating the spirit of its rules. Apprenticeship was usually delayed, and the hospital course made coincident with the last three years of the nominal servitude; an arrangement by which much objectionable drudgery was done away with, and by which three precious years were gained for school. But the fact remained, that boys of sixteen could never be sufficiently prepared, in general, for the reception of special education. They were cast into the ranks of a profession which, of all others, most requires a trained and philosophic mind, at an age when their minds were untrained and chaotic. Some passed safely through the ordeal, and emerged Abernethys or Coopers. The great majority became bewildered by complicated problems, with which they had never been trained to grapple, and limited their views of causation to the last antecedent; of effect, to the last consequent. They were developed by time into the class of practitioners who constitute the weakness of the profession, and who afford whatever basis of truth there may be for the attacks of satirists.

Intellectual and moral weakness are very commonly coincident, and a system of training that seemed intended to prevent young men from being logical, could scarcely fail to render them indifferent to obligations higher than those of science. According to our observation, the men upon whom apothecaryism has fallen with its full weight, are affected injuriously both in mind and conscience. Permitted or encouraged during apprenticeship to undertake medical duties empirically, and without the knowledge necessary for their due discharge, they learn to aim at the end without securing the means, and to be content with a diagnosis and treatment founded upon guesswork. In this way, they gradually ignore the fact that they cannot, without grave delinquency, neglect the opportunities of study afforded them; and they look forward to their examinations as ordeals to be got through anyhow; satisfied with themselves if they can by any means satisfy their questioners; and feeling no reproach when forced to convict themselves of ignorance. In practice, men of this class are found to fulfil the promise of their early lives, and to undertake the treatment of any case promising to be remunerative, whether they understand it or not; while among the poor they will seldom be at the trouble of investigation. We were once standing in the surgery of one of these worthies, when a poor man came in, having organic visceral disease stamped upon his countenance. His narrative of symptoms was speedily cut short, not by inquiries pertinent *ad rem*, but by "Give him some powders, Mr. Jones." Mr. Jones, the assistant, thrust his hand into a drawer full of powders, and with rapid and dexterous movement tied six of them into a packet. "Take one night and morning," he said, and the patient withdrew. When he was gone, we inquired of the principal, "What is the matter with that man?" "Oh, I don't know, I'm sure," was the reply; "I never saw him before. He is only a *club patient!*" "Well, but what did you give him?" "Oh, those powders. They contain some nitre, and a little Dover's powder, I think; don't they, Mr. Jones? We keep them for club patients." For such results as these, it is hard to say who are most to blame, the clubs or the apothecaries; but we incline to think the latter, because they have so long degraded the art of healing into a trade, that the public take them at their own valuation, and apply trade maxims to them. However this may be, it is quite evident that a system of education which places manual dexterity in compounding above cultivation of the intellect must be disastrous in its results, and must train up men having no proper consciousness of the importance of their calling, or of the heavy moral and intellectual responsibilities imposed by the confidence vested in them. And although medical students never were, and never could be, the yahoos depicted by Mr. Smith,—still medical students have, by their shortcomings, set their seal upon one generation of the profession. To them it is due, we think, that medical men although marching have not kept pace with the times, and that while advancing, they have retrograded relatively to the advance of other classes—such as the clergy and the legal profession. Quackery of all kinds is mainly due to professional errors; and although fashionable quackery may be due to errors arising out of the present state of science, or resulting necessarily from human infirmity, this is but a small portion of the whole. The quackery that affects the million, and that too often saps the health and strength of the poor, the stall of the medicine-vendor in the country market-place, his handbills and house-to-house visitation in the village, and the counter practice of druggists in towns, all these are due to professional errors arising out of the carelessness and culpable ignorance of students who have neglected the opportunities of acquisition afforded them. The practice of selling medicine, of representing it as the element of value in medical attendance (as if a carpenter should charge for his tools instead of his labour), has also greatly aided the impositions of the quack, who ever bases his pretensions upon his nostrum rather than upon his skill.

To culpable and avoidable professional errors we ascribe another remarkable feature of the present age in medicine—that is to say, the existence of special hospitals. They represent, not the knowledge of the few, but the ignorance of the many—the dishonest ignorance, that tries to pass itself off for knowledge, and that undertakes duties for which it has never been at the pains to qualify itself. Whether or not it be possible for ordinary practitioners to become thoroughly skilful, with hand, senses, and judgment, in every branch of the profession, so that a case of talipes or of ophthalmia will eventually be as well treated by the parish doctor as it would be in Oxford-street or at Moorfields, is a question that only time can solve. But we have seen double congenital talipes mistaken for paraplegia, until the patient, at the age of fifteen, was accidentally seen by a "special" practitioner; and we have seen, during a single morning visit to a provincial ophthalmic hospital, at least a dozen persons who had been positively blinded by injurious, or allowed to become blind by misdirected treatment. Correal ulcers had been doctored by lead lotions, so as to leave indelible cicatrices; and ophthalmia, produced by the irritation of eyelashes, or other foreign substances, had been assailed by some of the most potent weapons in the medical armoury, while the cause of the mischief remained undiscovered and unremoved.

We trust, however, that the session now commencing will be the precursor of a new and better state of things. Under the provisions of the Medical Act, there will be no necessity for general practitioners to possess the Apothecaries' licence, or to undergo the Apothecaries' apprenticeship. The recommendation of the Council, that all students shall be required to give evidence of having received a liberal education prior to the commencement of medical study, will serve to keep young men at school or college until they are old enough to go to a hospital, and while it will greatly raise the standard of mental cultivation, the expense entailed will serve indirectly to raise also the standard of social station, and to fill the lecture-rooms with men having the feelings of gentlemen, rather than the desires of traders. And it behoves the new and higher class of students, promised to us for the future, to lay seriously and earnestly to heart the responsibilities which their advantages entail. Their predecessors, notwithstanding the heavy yoke of the Hall, have, in the great majority of instances, shaken themselves free from the results of bearing it. Here and there, it is true, the typical apothecary may be found, ignorant, careless, greedy, venal; neglecting his patients for his ease, or abusing their confidence for his profit, and casting the shadow of his character over the acts and opinions of wiser and better men. But the great bulk of the practitioners of England, both in point of intellectual and moral character, deserve praise which can only be fairly meted by those who know the hindrances of student life; and we warn our young friends, now about to plant their feet upon that path, which, having travelled over, we cannot look back upon without regret, that the public have a right to expect great things from them. "Art is long, and life is fleeting;" the time allotted to medical study is fleeting indeed, and every portion of it that is wasted in idleness, or consumed in vice, will not only be the parent of bitter, unavailing regret in the face of the responsibilities of the future, but will most surely tend to degrade the medical body in the degradation of the individual delinquent. We expect, then, that our educated students shall show a right appreciation of their opportunities and of their duties, that they shall bring to the dissecting-room and the lecture-hall minds accustomed to reflection, and habits obedient to the voice of conscience. In other departments of life, men may be idle or careless with less of culpability, with the prospect, at least, of being themselves the chief, if not the only sufferers. In medicine, the case is widely different, for the errors of the ignorant practitioner fall upon the defenceless, and are often irremediable. From errors of some kind, no man, however skilful or learned, can hope to be entirely exempt; but such evidences of human frailty will become more rare just in proportion as our ranks are recruited by students of enlarged views and cultivated intellects. Empirical practice, notions of applying specific formulæ for the cure of nosological distinctions, will vanish before a system based upon scientific pathology and rational therapeutics; and miserable quarrellings, envyings, jealousies, bickerings, would be impossible among men accustomed to practise instinctive courtesy, to respect each other because respecting themselves, and to be ruled by an unwritten code of ethics, arising out of a clear comprehension of their duties to their patients, to the public, and to their brethren.

